# Supplementary material for: Creating Locally-Resolved Mobile-Source Emissions Inputs for Air Quality Modeling in Support of an Exposure Study in Detroit, Michigan, USA
Source: Int J Environ Res Public Health. 2014 Dec 9;11(12):12739–66. doi: 10.3390/ijerph111212739 (PMC4276644; doi:10.3390/ijerph111212739)
Supplement: Supplementary File 1 [file ijerph-11-12739-s001.pdf]

Supplementary Information

Creating Locally-Resolved Mobile-Source Emissions Inputs for Air Quality Modeling in Support of an Exposure Study in Detroit, Michigan, U.S.A.

Table S1. Acronyms used in this manuscript.

Acronym	Description
AADT	Average Annual Daily Traffic
CAADT	Commercial Average Annual Daily Traffic
DET	Detroit City Airport
EF	Emission Factor
EPA	Environmental Protection Agency
FHWA	Federal Highway Administration
HD	High Diesel
HDDV	Heavy-Duty Diesel Vehicles
HDGV	Heavy-Duty Gasoline Vehicles
HPMS	Highway Performance Monitoring System
HTHD	High Traffic High Diesel
HTLD	High Traffic Low Diesel
LD	Low Diesel
LDDT	Light-Duty Diesel Trucks
LDDV	Light-Duty Diesel Vehicles
LDGT1	Light-Duty Gasoline Trucks1
LDGT2	Light-Duty Gasoline Trucks2
LDGV	Light-Duty Gasoline Vehicles
LT	Low Traffic
MC	Motorcycles
MDOT	Michigan Department of Transportation
MOVES	Motor Vehicle Emissions Simulator
NEXUS	Near-Road Exposure and Effects of Urban Air Pollutants Study
NFC	National Functional Class
NWS	National Weather Service
PTR	Permanent Traffic Recorders
R-LINE	Research Dispersion Model for Line Sources
SEMCOG	Southeast Michigan Council of Governments
TAF	Temporal Allocation Factor
TDM	Travel Demand Model
VMT	Vehicle Miles Traveled

Figure S1. National functional class (NFC) designations for the Detroit area.

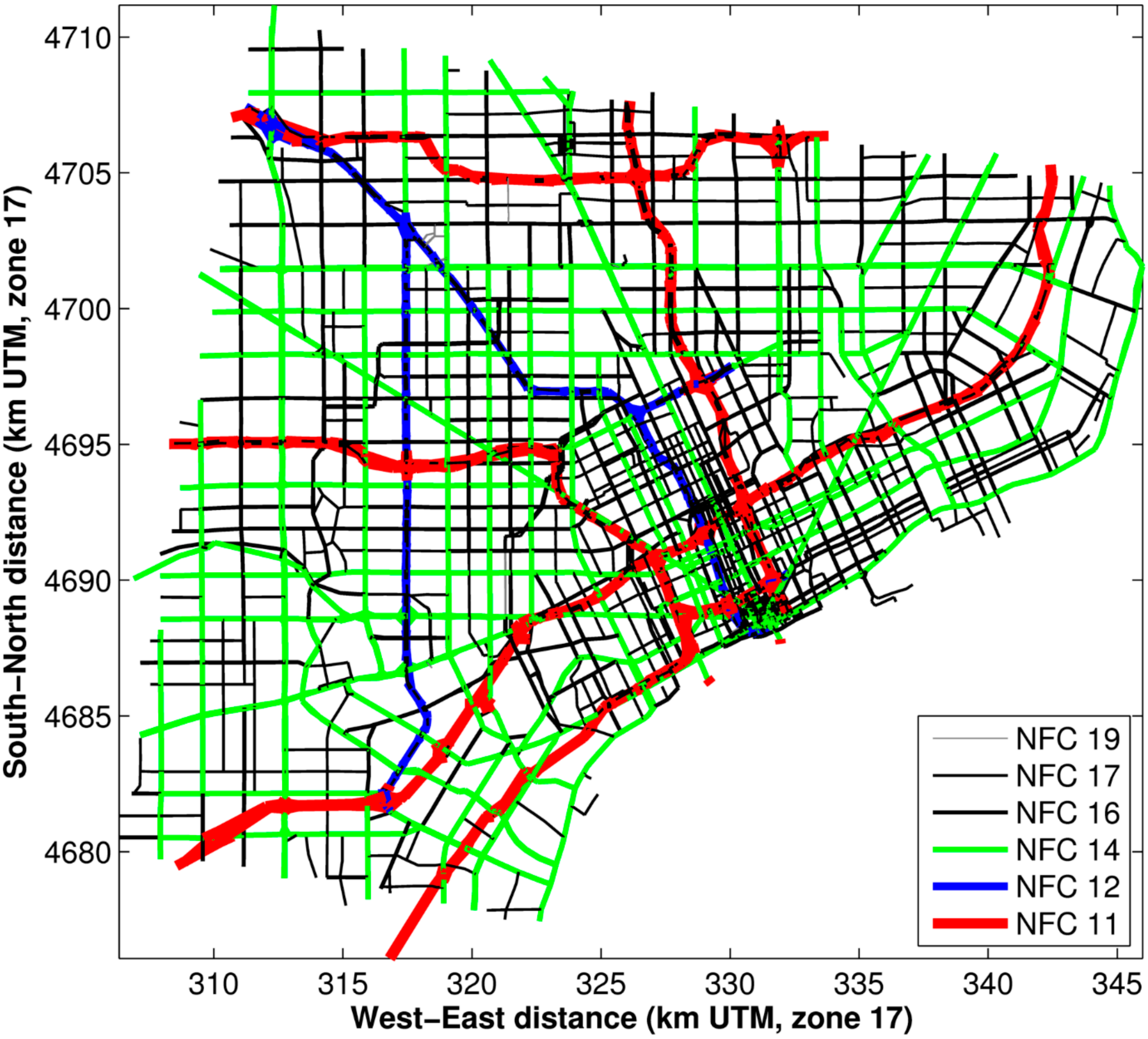

Figure S2. Temporal allocation factors (TAFs) for time of day (weekday: top left, weekend: top right), day of week (bottom left), and month of year (bottom right).

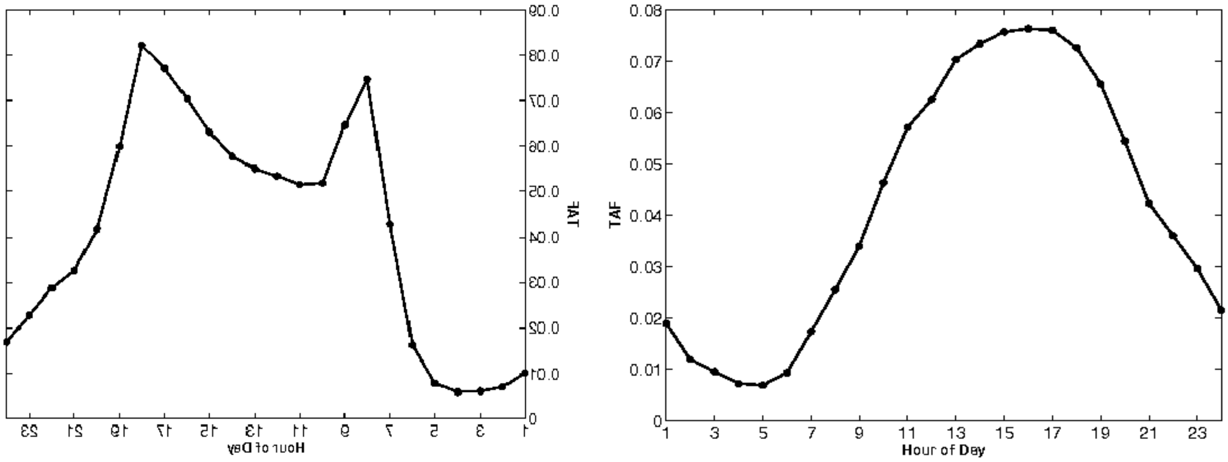

Figure S2. *Cont.*

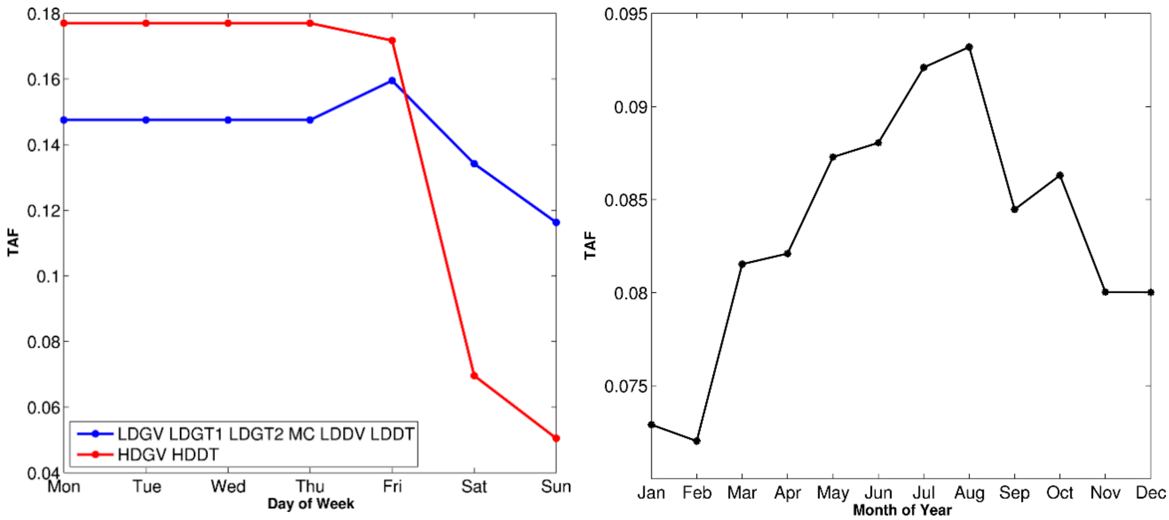

Figure S3. Emissions of $PM_{2.5}$, NO_x, and CO as a function of speed for August 2010 for a temperature of 55 °F.

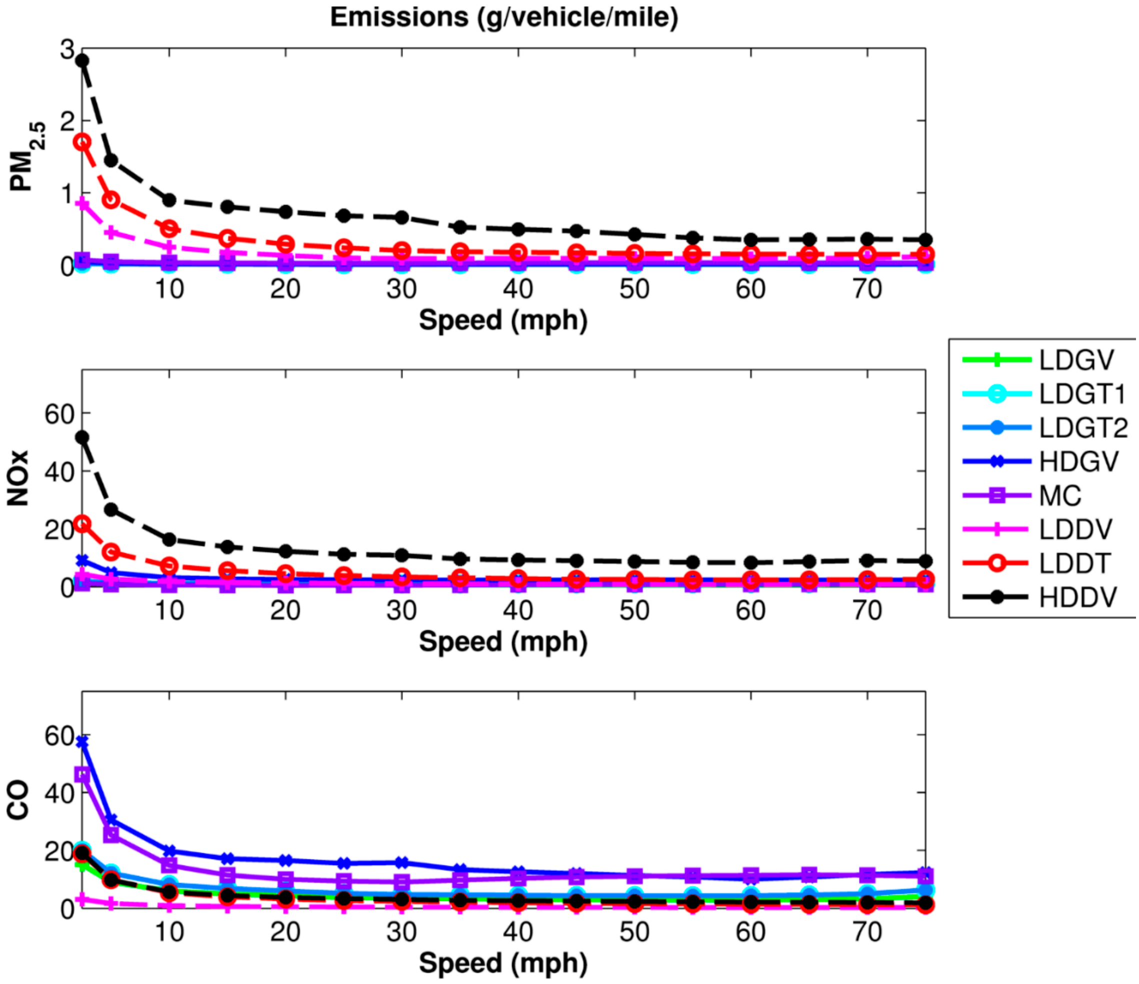

Figure S4. Emissions of $PM_{2.5}$, NO_x, and CO as a function of temperature for August 2010 for a speed of 45 mph.

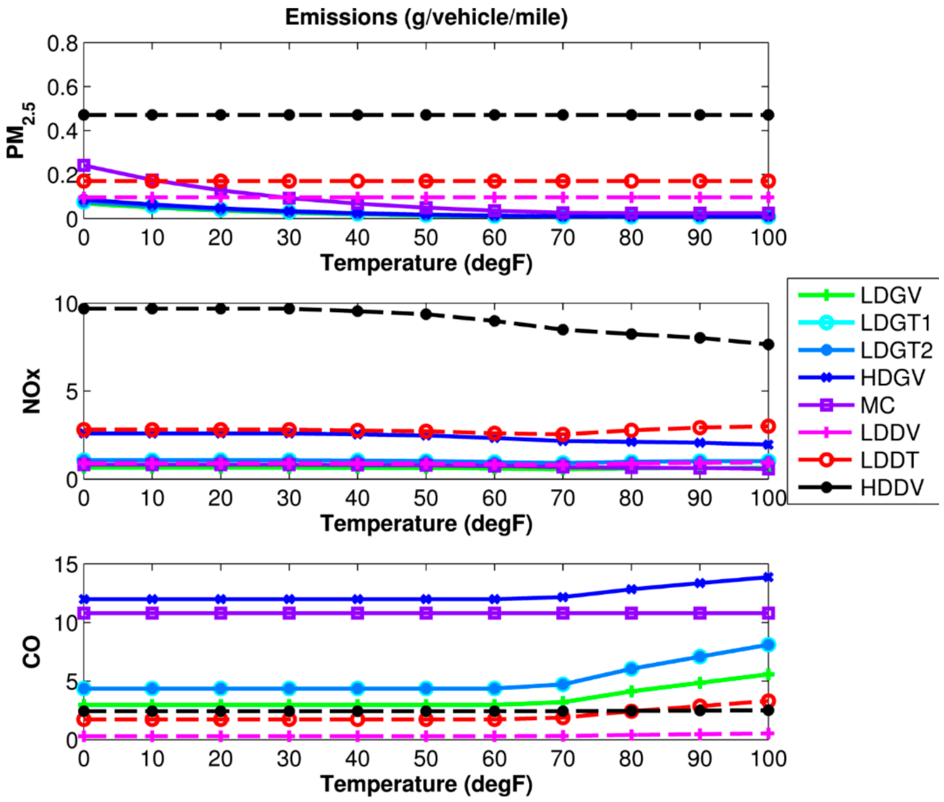

Figure S5. Locations of PTR measurements by id in Detroit, MI, USA.

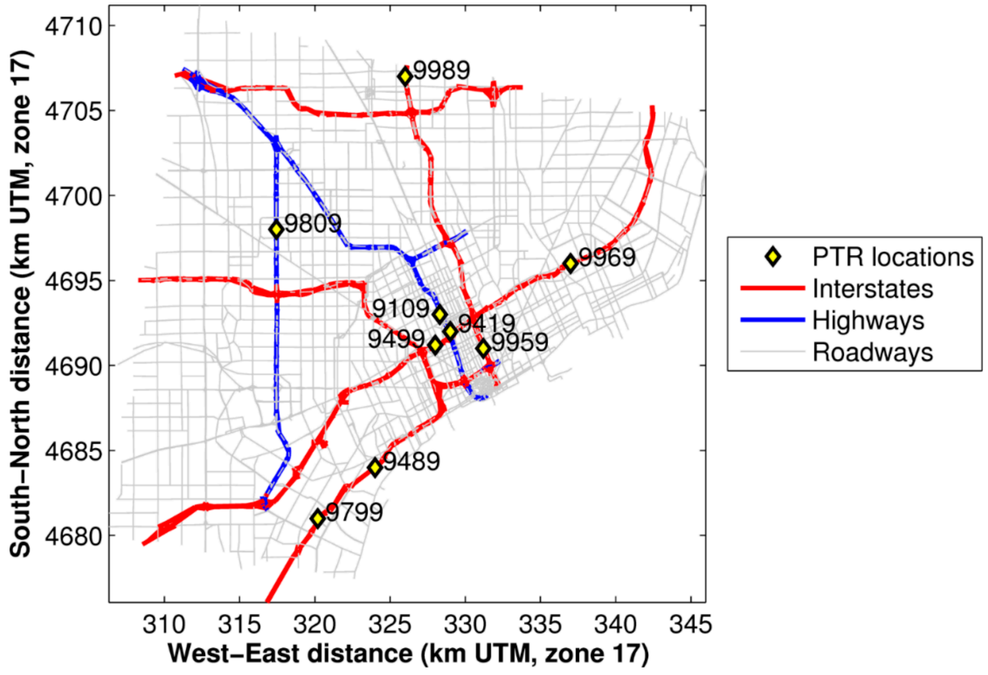

© 2014 by the authors; licensee MDPI, Basel, Switzerland. This article is an open access article distributed under the terms and conditions of the Creative Commons Attribution license (http://creativecommons.org/licenses/by/4.0/).
